# The effect of environmental change on vascular plant and cryptogam communities from the Falkland Islands and the Maritime Antarctic

**DOI:** 10.1186/1472-6785-7-15

**Published:** 2007-12-19

**Authors:** Stef Bokhorst, Ad Huiskes, Peter Convey, Rien Aerts

**Affiliations:** 1 Netherlands Institute of Ecology, Centre for Estuarine and Marine Ecology, Korringaweg 7, 4401 NT Yerseke, The Netherlands; 2 British Antarctic Survey, Natural Environmental Research Council, High Cross, Madingley Road, Cambridge CB3 0ET, UK; 3 Institute of Ecological Science, Department of Systems Ecology, Vrije Universiteit, De Boelelaan 1085, 1081 HV Amsterdam, The Netherlands; 4 Department of Animal and Plant Sciences, University of Sheffield, Western Bank, Sheffield, S10 2TN, UK

## Abstract

**Background:**

Antarctic terrestrial vegetation is subject to one of the most extreme climates on Earth. Currently, parts of Antarctica are one of the fastest warming regions on the planet. During 3 growing seasons, we investigated the effect of experimental warming on the diversity and abundance of coastal plant communities in the Maritime Antarctic region (cryptogams only) and the Falkland Islands (vascular plants only). We compared communities from the Falkland Islands (51°S, mean annual temperature 7.9°C), with those of Signy Island (60°S, -2.1°C) and Anchorage Island (67°S, -2.6°C), and experimental temperature manipulations at each of the three islands using Open Top Chambers (OTCs).

**Results:**

Despite the strong difference in plant growth form dominance between the Falkland Islands and the Maritime Antarctic, communities across the gradient did not differ in total diversity and species number.

During the summer months, the experimental temperature increase at 5 cm height in the vegetation was similar between the locations (0.7°C across the study). In general, the response to this experimental warming was low. Total lichen cover showed a non-significant decreasing trend at Signy Island (p < 0.06). In the grass community at the Falkland Islands total vegetation cover decreased more in the OTCs than in adjacent control plots, and two species disappeared within the OTCs after only two years. This was most likely a combined consequence of a previous dry summer and the increase in temperature caused by the OTCs.

**Conclusion:**

These results suggest that small temperature increases may rapidly lead to decreased soil moisture, resulting in more stressful conditions for plants. The more open plant communities (grass and lichen) appeared more negatively affected by such changes than dense communities (dwarf shrub and moss).

## Background

Antarctica is the coldest, driest, windiest and highest continent on Earth. As a result, plant growth is largely limited to the coastal areas of the sub- and maritime-Antarctic regions. In these regions, there are small areas where vascular plants and cryptogams (mosses and lichens) can grow due to the summer melt of snow and ice. Due to the harsh climate, Antarctic vegetation mainly consists of cryptogams and there are only two vascular plant species (*Deschampsia antarctica *and *Colobanthus quitensis*). The extreme environmental conditions provide one of the main reasons why ecosystems in the Antarctic regions are relatively simple, have a poorly developed trophic structure and are species poor as compared to lower latitude ecosystems [1,2].

Recent climate changes documented over the last 50 years along the Antarctic Peninsula have been far greater than seen at lower latitudes. The most conspicuous changes have been an average increase in temperature of about 2°C over this period [3], and changes in patterns of cyclonic activity around the Antarctic continent, along with changes in precipitation intensity [4]. The warming trends along the Antarctic Peninsula are not constant throughout the year, with higher increases during winter than during the summer [3].

These changes raise the question of whether this (small) apparent reduction in ecological stress through warming will be sufficient to influence the distribution and abundance of cryptogams and vascular plants in this harsh environment. There are already some indications that this is the case for some species, as climatic changes have been implicated in affecting the local population density and distribution of the two vascular plant species (*Deschampsia antarctica *and *Colobanthus quitensis*) that occur along the Antarctic Peninsula. In addition, their reproductive patterns have also changed, with a greater incidence of successful sexual reproduction and increased seed output [5-9]. Invasion of new species and shifts in species composition of communities are also predicted to occur [10-12] but yet to be observed.

Longer periods above biological thresholds are likely to affect the life cycle of cryptogams in the context of growth rates, development and reproduction [13]. Warming of the soil and air may therefore increase the abundance and cover of mosses and lichens due to its influences on these life history traits [14-16]. However, warming may also influence water availability which might have a negative effect on cryptogam development depending on the rate of evaporation or melting of snow and ice. Arctic studies have shown that cryptogams can decrease in abundance and biomass due to warming [17-21], in most cases as a result of increased cover and competition from vascular plants [22]. As vascular plants are very restricted in the Antarctic, the main response here to increased temperatures will be one of the cryptogams alone.

**Figure 1:**
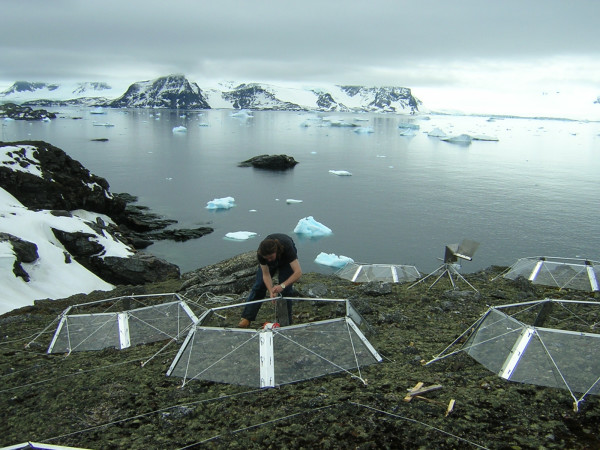
OTC in situ at Signy Island.

We studied the responses of Maritime Antarctic plant communities to temperature change by using two complementary methods: (1) Studies at three field locations, ranging from the cold temperate oceanic Falkland Islands to southern Maritime Antarctic Anchorage Island, spanning a natural latitudinal and environmental gradient. This 'climate change by location substitution' can be seen as a proxy for the possible long-term effect of warming on the Antarctic vegetation. In this context, we consider the Falklands Islands as an analogue for extremely warmed Maritime Antarctic Islands. (2) Investigations using multi-year field experiments at each of the three locations, where temperature was experimentally manipulated using Open Top Chambers (OTCs) (Fig. 1) [23].

## Results

### Environmental data

**Figure 2:**
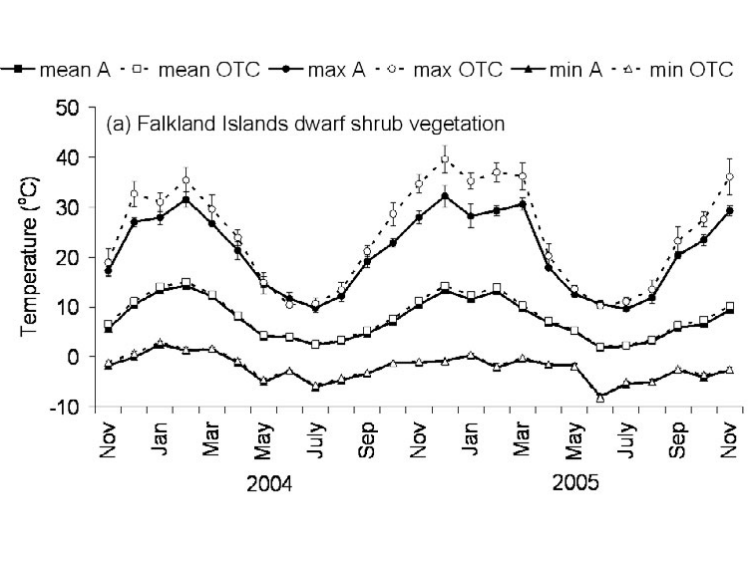
**Temperature graph of dwarf shrub vegetation on the Falkland Islands**. The monthly mean, maximum and minimum temperature at 5 cm above the soil in the control and OTC plots of the dwarf shrub vegetation on the Falkland Islands. A: ambient temperature in control plots, OTC: Temperature in OTC. n = 3 for each monthly value, error bars indicate se. Data represent period between November 2003 and November 2005.

**Figure 3:**
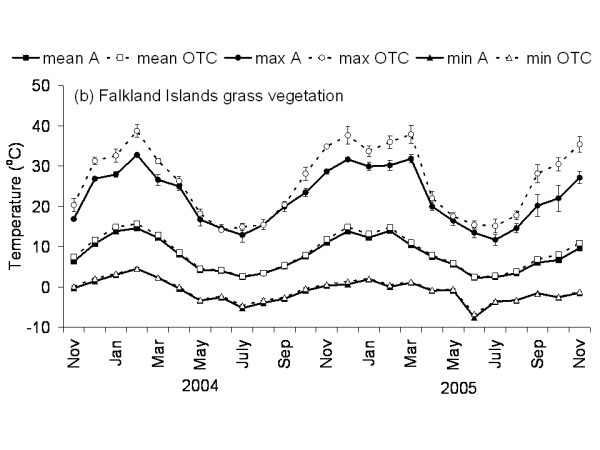
**Temperature graph of grass vegetation on the Falkland Islands**. The monthly mean, maximum and minimum temperature at 5 cm above the soil in the control and OTC plots of the grass vegetation on the Falkland Islands. A: ambient temperature in control plots, OTC: Temperature in OTC. n = 3 for each monthly value, error bars indicate se. Data represent period between November 2003 and November 2005.

**Figure 4:**
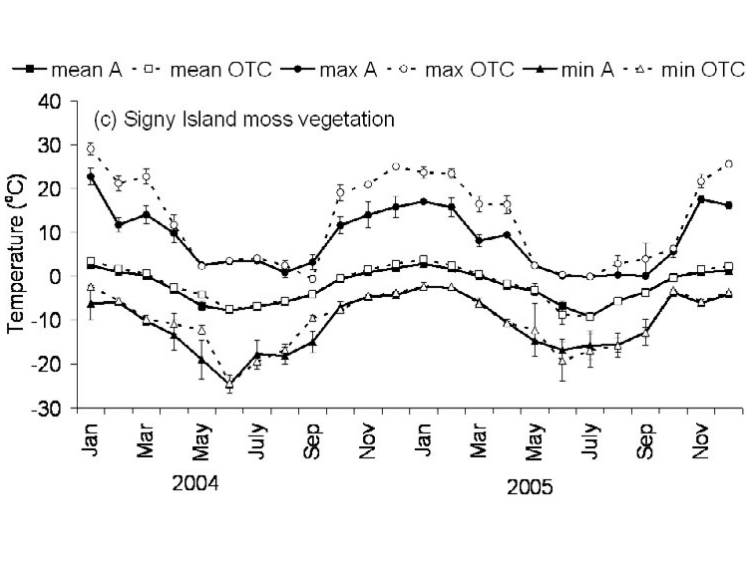
**Temperature graph of moss vegetation on Signy Island**. The monthly mean, maximum and minimum temperature at 5 cm above the soil in the control and OTC plots of the moss vegetation on Signy Island. A: ambient temperature in control plots, OTC: Temperature in OTC. n = 3 for each monthly value, error bars indicate se. Data represent period between November 2003 and November 2005.

**Figure 5:**
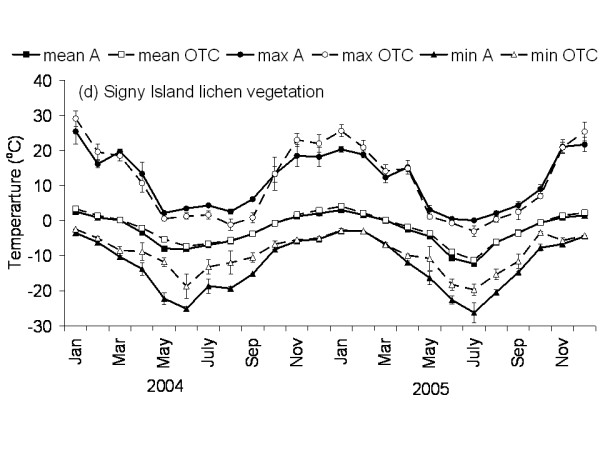
**Temperature graph of lichen vegetation on Signy Island**. The monthly mean, maximum and minimum temperature at 5 cm above the soil in the control and OTC plots of the lichen vegetation on Signy Island. A: ambient temperature in control plots, OTC: Temperature in OTC. n = 3 for each monthly value, error bars indicate se. Data represent period between November 2003 and November 2005.

**Figure 6:**
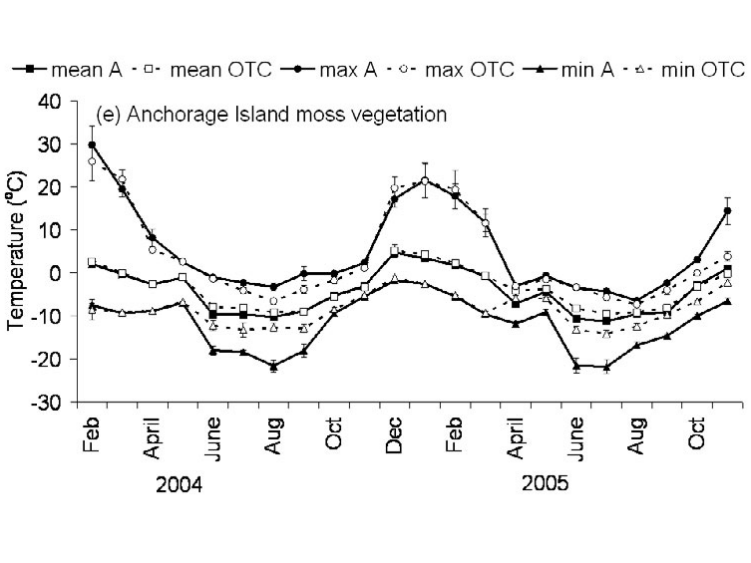
**Temperature graph of moss vegetation on Anchorage Island**. The monthly mean, maximum and minimum temperature at 5 cm above the soil in the control and OTC plots of the moss vegetation on Anchorage Island. A: ambient temperature in control plots, OTC: Temperature in OTC. n = 3 for each monthly value, error bars indicate se. Data represent period between November 2003 and November 2005.

**Figure 7:**
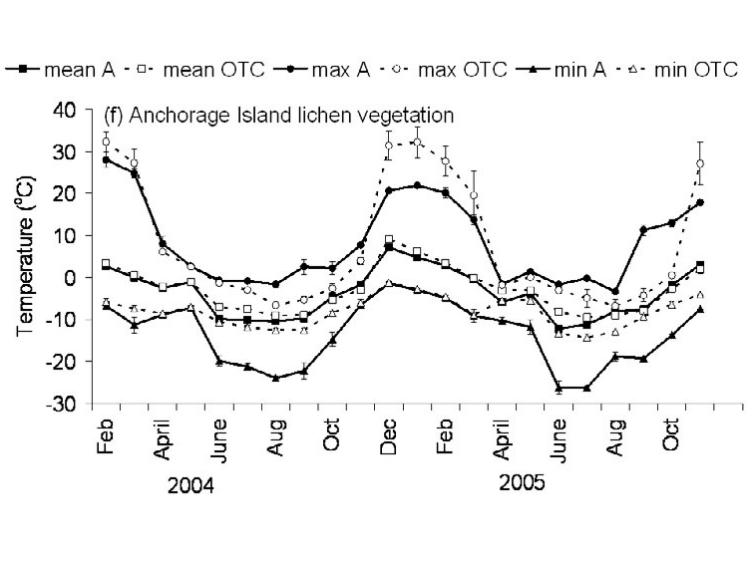
**Temperature graph of lichen vegetation on Anchorage Island**. The monthly mean, maximum and minimum temperature at 5 cm above the soil in the control and OTC plots of the lichen vegetation on Anchorage Island. A: ambient temperature in control plots, OTC: Temperature in OTC. n = 3 for each monthly value, error bars indicate se. Data represent period between November 2003 and November 2005.

**Figure 8:**
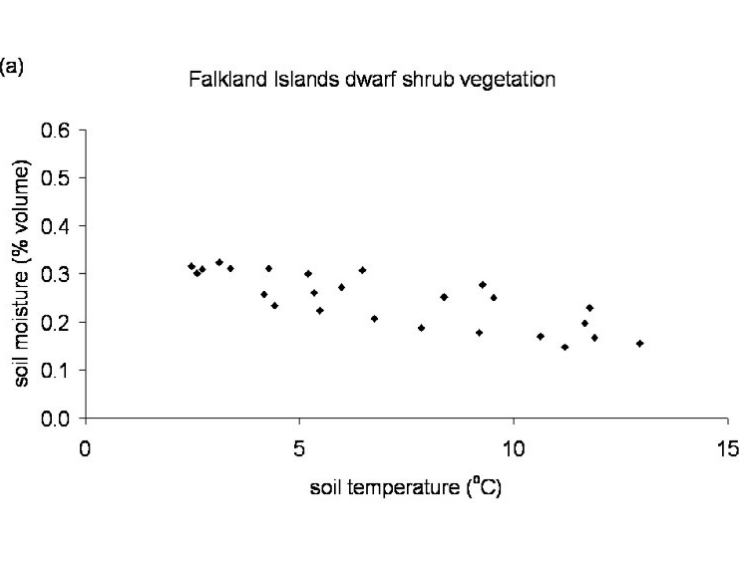
**Temperature soil moisture relationship Falkland Islands**. Relation between soil temperature and soil moisture in the dwarf shrub vegetation on the Falkland Islands. Data points represent monthly mean values of the period November 2003 till November 2005 from the control plots.

**Figure 9:**
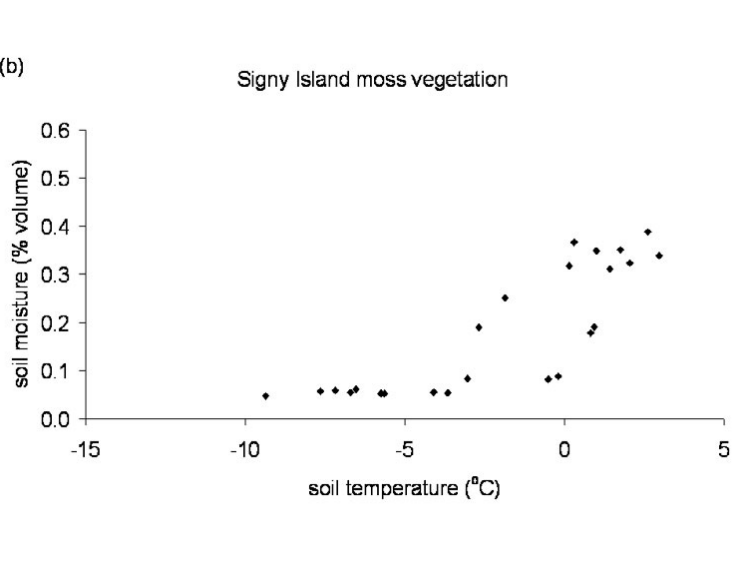
**Temperature soil moisture relationship Signy Island**. Relation between soil temperature and soil moisture in the moss vegetation on Signy Island. Data points represent monthly mean values of the period November 2003 till November 2005 from the control plots.

**Figure 10:**
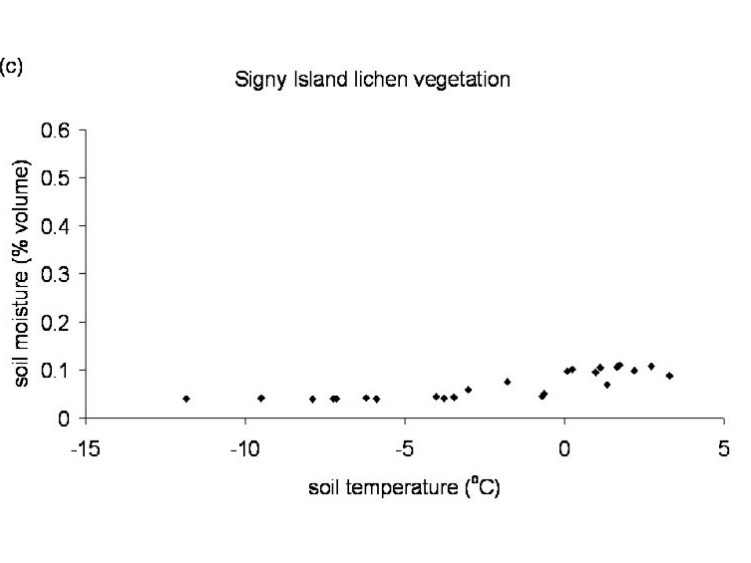
**Temperature soil moisture relationship Signy Island**. Relation between soil temperature and soil moisture in the lichen vegetation on Signy Island. Data points represent monthly mean values of the period November 2003 till November 2005 from the control plots.

**Figure 11:**
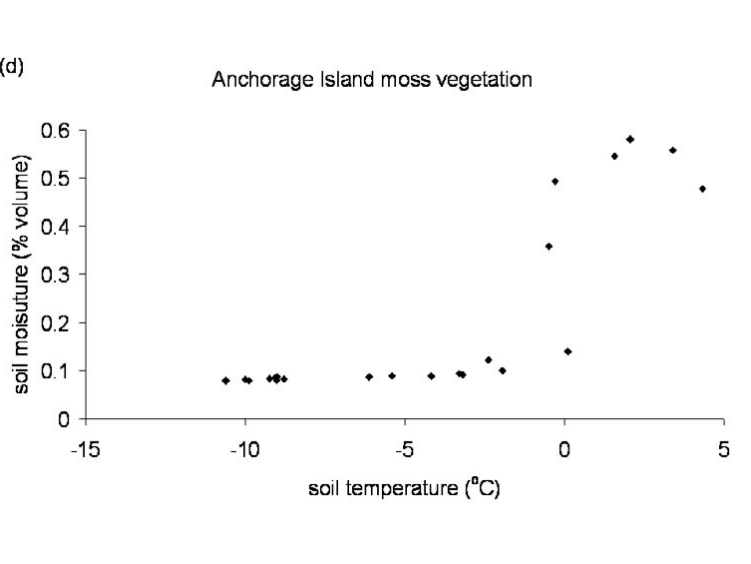
**Temperature soil moisture relationship Anchorage Island**. Relation between soil temperature and soil moisture in the moss vegetation on Anchorage Island. Data points represent monthly mean values of the period November 2003 till November 2005 from the control plots.

The Falkland Islands was the warmest of the three locations with the highest temperature, measured at 5 cm above the soil surface. Signy and Anchorage Island had lower and similar annual average temperatures. However, Signy Island had a lower summer temperature than Anchorage Island (Figures 2, 3, 4, 5, 6, 7). At the Falkland Islands, there was a negative relationship between soil temperature and soil moisture (r^2 ^= 0.64, *P *< 0.001, n = 25) in the dwarf shrub community (Fig. 8). On Signy (Fig. 9 and 10) and Anchorage Island (Fig. 11) the relation between temperature and soil moisture was non-linear. Therefore, we had to apply a generalised additive model with a Gaussian distribution. When the soil was deep frozen, the measured soil moisture approached zero, but when the temperature rose above -3.0°C, there was a linear increase in soil moisture with increasing temperature (r^2 ^= 0.70, *P *< 0.001, n = 24). Warming treatment had no effect on this relationship. Total rainfall during the 2004/05 year was 9% lower than the average (575 mm y^-1^) at the Falkland Islands (data obtained from the Falkland Islands Department of Agriculture). The main difference in rainfall occurred during February, with 30 mm less than average.

### Effects of OTC deployment

Table 1 summarises the effects of OTC deployment on temperature. Annual mean temperature increase at 5 cm height was on average 0.7°C (*P *< 0.05) higher in OTCs in all communities. However, there were differences between seasons and communities in the amount of warming achieved. Maximum and minimum temperatures were differently affected between the communities during summer and winter months as shown in Table 1 and Fig. 2, 3, 4, 5, 6, 7. Table 2 summarises the effect of OTC deployment on PAR, soil moisture and relative humidity. PAR was reduced (p < 0.05) in the OTCs on a yearly basis only. Soil moisture, based on TDR measurements, was reduced (p < 0.05) in the OTCs during summer and annually. Gravimetric determination of soil moisture indicated a marginally (*P *< 0.06) lower water value in the OTCs (26.7 ± 2.2 vs. 17.9 ± 2.5) of the grass community at the Falkland Islands. Relative humidity was reduced (P < 0.01) in OTCs only during summer.

**Table 1:** Effect of Open Top Chambers on air temperature

	**Temperature difference (OTC-Control)** **(°C)**							**Degree days** (sum >0°C)	
	at 5 cm					Maximum	Minimum		
	Summer	Autumn	Winter	Spring	Annual	Summer	Winter	Difference	% of control
**Falkland Islands**									
**grass**	0.9*	0.4'	0.3'	1.1*	0.7*	5.2 **	ns	235 *	9
**dwarf shrub**	0.9*	0.4'	0.2'	0.7*	0.6*	5.8 **	ns	195 *	8
**Signy Island**									
**lichen**	0.8**	0.6**	1.0*	0.2'	0.7**	ns	5.3 *	103 **	31
**moss**	0.8**	0.4**	-0.7*	0.1'	0.3**	6.0 **	ns	148 **	49
**Anchorage Island**									
**lichen**	1.2**	0.8**	2.6*	-0.8'	1.1**	9.5 **	10.3 *	80 **	21
**moss**	0.6**	0.7**	1.9*	-0.3'	0.8**	ns	6.8 **	26 **	10
' p < 0.1 * p < 0.05, ** p < 0.01									

Mean temperature, summer maximum, winter minimum and cumulative degree day differences between OTCs and control plots at 5 cm above the soil surface. Analyses based on the mean hourly data obtained between December 2004 and November 2005 for each community. Significant differences between OTCs and control plots are indicated. Degree days were calculated with 0°C as threshold as this is the most commonly used threshold for this variable. % of control indicates the percent increase in degree days in the OTC compared to the control value.

**Table 2:** Effect of Open Top Chambers on light, soil moisture and humidity

**Community**	**PAR**		**Soil moisture**		**Relative humidity**	
	Annual	Summer	Annual	Summer	Annual	Summer
	(Difference between control plots and OTCs as percentage)					
**Falkland Islands**	-7.3 *	-5.4				
**grass**	(-9.6)	(-8.7)	-	-	-3.5	-5.9 **
**dwarf shrub**	(-4.8)	(-2.1)	-20.2 *	-12.3**	-2.0	-4.3 **
**Signy Island**	-12.3 *	-7.1				
**lichen**	(-5.5)	(2.6)	-9.9 *	-20.1**	-2.7	-2.5 **
**moss**	(-19)	(-16.2)	-4.5 *	-8.8 **	-1	-1.3 **
**Anchorage Island**	-28.1 *	-9.2				
**lichen**	(-31.7)	(-9.6)	-	-	1.7	-3.1 **
**moss**	(-22.8)	(-8.9)	-2.8 *	-7.5 **	1.2	1.8 **
* p < 0.05, ** p < 0.01						

Effect of OTC deployment on photosynthetic active radiation (PAR) (n = 2 for each location but only 1 for each community), soil moisture and relative humidity as a percentage of the control value based on annual and summer means. The PAR values for each location are the means of the two values between brackets, indicating communities.

### Vegetation composition

Tables 3, 4 and 5 list the species present in the study plots at each location. Both at Signy and Anchorage Island total species number was lower (*P *< 0.05) in the moss communities (3 ± 1) than the lichen communities (7 ± 1). However, they did not differ between the grass and the dwarf shrub communities at the Falkland Islands (Table 6). Species composition (expressed as evenness) did not differ between communities (mean of 0.7 ± 0.1). At the Falklands, vegetation cover in the dwarf shrub community (95 ± 1%) was higher (*P *< 0.001) than in the more open structured grass community (39 ± 5%). Moss communities at respectively Signy and Anchorage Island had a higher (*P *< 0.001) total cover (99 ± 0 and 83 ± 5%) than the more open structured lichen communities (87 ± 3 and 57 ± 6 %). Despite the clear difference in growth form dominance, there were no significant differences in overall species diversity between the communities. All had a diversity index of around 1.0 (Table 6).

**Table 3:** Vegetation composition at the Falkland Islands study sites

	**Falkland Islands**			
**Vegetation type**	Dwarf shrub		Grass	
		(%)		(%)
(v): vascular plant				
	*Empetrum rubrum *(v)	68.3	*Poa annua *(v)	25.6
	*Festuca magellanica *(v)	22.0	*Festuca magellanica *(v)	15.7
	*Pernettya pumila *(v)	11.3	*Azorella caespitosa *(v)	6.3
	*Oxalis enneaphylla *(v)	8.0	*Colobanthus quitensis *(v)	4.7
	*Blechnum penna-marina *(v)	6.8	*Poa pratensis *(v)	4.1
	*Azorella lycopodioides *(v)	2.8	*Aira praecox *(v)	2.2
	*Trisetum spicatum *(v)	1.9	*Azorella filamentosa *(v)	<1.0
	*Azorella caespitosa *(v)	1.7		
	*Acaena lucida *(v)	<1.0		
	*Cortaderia pilosa *(v)	<1.0		
	*Olsynium filifolium *(v)	<1.0		
	*Rubus geoides *(v)	<1.0		

Mean cover (%) of vascular plants in the control plots of each vegetation type at the Falkland Islands at the start of the experiment. Species are listed in order of abundance from top to bottom.

**Table 4:** Vegetation composition at the Signy Island study sites

	**Signy Island**			
**Vegetation type**	Moss		Lichen	
		(%)		(%)
(l): lichen, (m): moss				
	*Chorisodontium aciphyllum *(m)	76.2	*Usnea antarctica *(l)	52.9
	*Polytrychum strictum *(m)	62.8	*Andreae depressinervis *(m)	20.4
	*Cladonia gracilis *(l)	8.8	*Ochrolechia frigida *(l)	12.0
	*Andreaea depressinervis *(m)	<1.0	*Buellia perlata *(l)	5.1
	*Ochrolechia frigida *(l)	<1.0	*Cladonia gracilis *(l)	4.5
	*Usnea antarctica *(l)	<1.0	*Polytrychum strictum *(m)	2.4
	*Sphaerophorus globosus *(l)	<1.0	*Sphaerophorus globosus *(l)	2.0
	*Alectoria nigricans *(l)	<1.0	*Alectoria nigricans *(l)	1.5
			*Cetraria aculeata *(l)	<1.0
			*Chorisodontium aciphyllum *(m)	<1.0

Mean cover (%) of cryptogams in the control plots of each vegetation type Signy Island at the start of the experiment. Species are listed in order of abundance from top to bottom.

**Table 5:** Vegetation composition at the Anchorage Island study sites

	**Anchorage Island**			
**Vegetation type**	Moss		Lichen	
		(%)		(%)
(a): alga, (l): lichen, (m): moss				
	*Sanionia uncinata *(m)	47.8	*Buellia latemarginata *(l)	30.0
	*Brachythecium austrosalebrosum *(m)	21.6	*Usnea antarctica *(l)	21.3
	*Prasiola crispa *(a)	19.0	*Rhizoplaca aspidophora *(l)	9.8
	*Pohlia nutans *(m)	11.4	*Acarospora macrocyclos *(l)	4.8
	*Buellia spp*. (l)	<1.0	*Prasiola crispa *(a)	1.8
	*Cephaloziella varians*	<1.0	*Buellia spp*. (l)	6.0
	*Usnea antarctica *(l)	<1.0	*Cephaloziella varians*	<1.0
	*Acarospora macrocyclos *(l)	<1.0	*Umbilicaria decussata *(l)	<1.0
			*Xanthoria elegans *(l)	<1.0

Mean cover (%) of cryptogams in the control plots of each vegetation type at Anchorage Island at the start of the experiment. Species are listed in order of abundance from top to bottom. (*Cephaloziella varians *is a liverwort)

**Table 6:** Plant diversity at the study sites and after warming

		**2003**	**H**	**H**	**H change (2005–2003)**	
**Locations**	**Community**	**nr species**	**Control**	**OTC**	**Control**	**OTC**
**Falkland Islands**						
	**grass**	5 ^ab^	0.8 (0.2)^a^	0.6 (0.2)	0.5 (0.1)	-0.0 (0.2)
	**dwarf shrub**	6 ^a^	1.1 (0.2)^ab^	1.1 (0.2)	-0.1 (0.1)	-0.2 (0.1)
**Signy Island**						
	**lichen**	7 ^a^	1.3 (0.1)^ab^	1.1 (0.2)	-0.1 (0.1)	-0.1 (0.1)
	**moss**	3 ^b^	0.8 (0.1)^a^	0.7 (0.0)	-0.0 (0.0)	-0.0 (0.0)
**Anchorage Island**						
	**lichen**	8 ^a^	1.5 (0.1)^b^	1.3 (0.1)	-0.3 (0.1)	-0.5 (0.1)
	**moss**	3 ^b^	0.6 (0.1)^a^	0.9 (0.1)	0.0 (0.1)	-0.1 (0.2)

Species number and diversity of the vegetation expressed in Shannon's diversity index (H). Different letters indicate significant (P < 0.05 Tukey HSD) differences in diversity between communities (tested for controls only as there was no effect after two years of warming).   Values between brackets are se.

**Figure 12:**
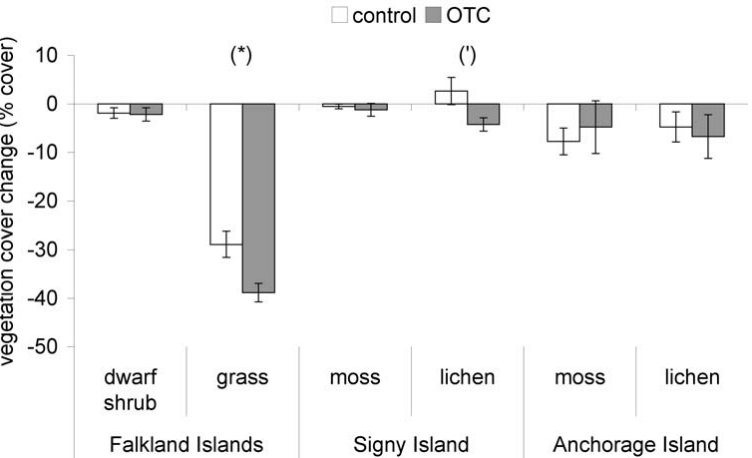
**Vegetation cover change after warming**. Total vegetation cover change, after 3 seasons of warming. n = 9 for the dwarf shrub and n = 3 for the grass communities on the Falkland Islands. The four communities at the Maritime Antarctic Islands all have n = 6. * indicates significant differences (p < 0.05 Tukey HSD) between OTC and control plots (') indicates a marginal difference (p < 0.1), error bars are se.

There was no change in vegetation diversity due to OTC deployment over the study period at any of the locations. The increase in diversity in the control plots of the grass community at the Falkland Islands (Table 6) was due to a shift in the evenness between species, which was higher (*P *< 0.05) in the control plots in 2005 (0.9 ± 0.02) than in 2003 (0.5 ± 0.07). Total vegetation cover, after two years of warming, was reduced in the grass community at the Falkland Islands (*P *< 0.05) and marginally reduced in the lichen (*P *< 0.06) community at Signy Island (Fig. 12), but no significant changes were apparent in the other communities at any location. At the individual species level no significant effects of the OTC deployment were detected. However, three species almost completely disappeared from both the control plots and OTCs over the course of the study in the Falkland Islands grass community. *Festuca magellanica *decreased by 91.0% in the control plots and 98.7% in the OTCs. *Poa annua *decreased by 98.7% and 100%, and *Aira praecox *by 87.5% and 100%, from the control plots and OTCs respectively. The lack of significance despite these apparently large changes was due to high variability and relatively low replication in this specific community (n = 3). At Signy Island and Anchorage Island, none of the individual species showed a significant response to the OTCs.

## Discussion

### Experimental design

To our knowledge, this is the first study in this region that has explicitly examined the response of the vegetation to experimental warming and compared this response across different communities at different latitudes. However, even though our three research locations were evenly spaced in terms of latitude, they were not spaced evenly in terms of the environmental conditions experienced. There was a much greater contrast between the Falkland Islands and the two Maritime Antarctic locations than there was between the latter. This difference is also reflected in the completely different native vegetation composition. However, despite these differences, total species diversity did not differ between the communities. The climatic differences between Signy Island and Anchorage Island are most likely related to the lower insolation at Signy Island [24].

We consider the Falkland Islands as a possible analogue for extremely warmed maritime Antarctic Islands. However, at present the plant species composition at the Falklands is completely different from that at the maritime Antarctic Islands. The almost complete absence of vascular plants in the maritime Antarctic locations can be attributed to the current climatic extremes and migration barriers for vascular plants [25-27]. As plants respond to climatic extremes rather than to climatic averages, the chances for successful establishment in a climate that is becoming warmer may be severely constrained by the occurrence of extreme conditions [28-30]. The recent colonisation of 'new' vascular plants on sub-Antarctic islands most likely has an anthropogenic cause [10,31]. These islands are the first stepping stones for new colonisers as the climate is less harsh than along the Antarctic Peninsula and the main continental landmass. Amelioration of the climate is expected to increase the chances for vascular plants to colonize the Antarctic Peninsula region. However, successful establishment will be hampered by the occurrence of extreme events such as droughts and summer freezing events.

### The effect of experimental warming on vegetation

Temperature and soil moisture were prominently affected by the OTCs. Relative humidity measures were slightly reduced during summer and a reduction in PAR receipt was detected on a yearly timescale only. This was most likely due to the lower zenith angle of the sun at these high latitudes. During the summer months, no effect of OTCs on PAR levels was apparent. Any important influences of OTCs on plant and cryptogam growth are therefore likely to be underlain by the warming experienced during the summer months and the reduction in soil moisture. These changes in the environmental conditions caused by the OTCs' for the vegetation resemble the current and predicted future changes for the Antarctic Peninsula region [32]. Although it has to be noted that precipitation changes are harder to predict. To counteract any soil water loss due to higher temperature, increasing precipitation would be required. However, the temporal variability of increased precipitation is even harder to predict and to study this effect, would require a far more elaborate experimental scheme, similar to that used by Dorrepaal *et al. *[15]. Given that the predicted temperature changes are the most reliable of Global Change Models, the use of the current OTCs allowed us to study the direct effects of a temperature increase, including increased evaporation, on the vegetation. In contrast to other methodologies, i.e., smaller and closed chambers, where temperature increases might have been more effective but resulted in unwanted side effects [23,33].

The more open structured communities (lichen and grass) appeared to be more responsive, in terms of temperature increase, to this passive warming than the more closed communities, which probably are better capable of buffering for such environmental perturbations. This is probably the reason why the strongest responses were found in these open structured communities. Total vegetation cover was negatively affected by OTCs' in the grass community at the Falkland Islands and the lichen community at Signy Island. Other than the study of Kennedy [34], the consequences of environmental change for lichen dominated vegetation have not been addressed experimentally in the Antarctic, while at Arctic sites most responses of lichen species to experimental warming have been attributed to the negative (competitive) consequences of increases in vascular plants [22]. As vascular plants were absent from our Antarctic study sites, other factors must underlie the response seen in these lichens. The carbon balance of Antarctic lichens is highly dependent on tissue moisture [35]. This suggests the possibility of drought stress due to the warming treatment at Signy Island, as reflected in the soil moisture and humidity data, and proposed in a different methodology screen manipulation study by Convey *et al.*[36]. That a similar response was not found at Anchorage Island might relate to the nature of the substratum – at Signy Island there is some development of a shallow layer of mineral soil which may provide some water holding capacity, while the lichens at Anchorage Island mainly grow directly on the rock substratum. The lichen species at Anchorage Island are perhaps adapted to more stressful environmental drought conditions and might therefore respond only after prolonged changes in environmental conditions. Increased drought stress is also likely to underlie the vegetation cover decrease seen in OTCs in the grass community of the Falkland Islands.

Both overall diversity measures and individual species data were not significantly affected by the OTCs. However, two grass species (*Poa annua *and *Aira praecox*) on the Falkland Islands did disappear within the OTCs after two years of warming. *Festuca magellanica *also almost disappeared from the OTCs but these three species also showed a very large decline in the control plots. The summer of 2004/05 was relatively dry compared to previous years on the Falkland Islands. Although this is probably not an extreme event for such ecosystems, in combination with the temperature increase it might have led to increased stress for these species. Therefore, germination during the 2005 spring may have been inhibited due to the previous 'drier' period [37,38] in combination with the extra warming.

Previous warming studies in the Arctic have stimulated increases in above ground vegetation biomass [9,39-42]. However, decreases and shifts in community composition as a result of warming have also been reported [14,22,43,44]. The small response of the communities examined in this study may be related to the small increase in mean temperatures achieved in the OTCs, and the relatively short duration of the experiment (even though few environmental manipulation studies involve continuous multi-season deployment). Also, the vegetation responses described in the previous studies focused mainly on vascular plants. In contrast, the current study focused solely on cryptogamic species at the Maritime Antarctic locations. These species have a much lower rate of growth compared to vascular plants, and measurable responses in abundance may need more time to develop than was available in the duration of this study [28]. That the dwarf shrub community on the Falkland Islands did not show any responses is most likely a result of the relative short duration, low temperature increase and perhaps dry microclimate of the community. Similar studies in the sub-Arctic generally do show responses in dwarf shrub communities after warming [39,40] but these generally generate a higher temperature increase, lasted longer and most likely had a different soil moisture regime. Nevertheless, the observation that the experimental manipulations during 3 growing seasons of the current study were sufficient to generate some detectable responses may indicate that these communities are susceptible to climate change over a longer time period.

## Conclusion

Our (short-term) results indicate that even small temperature increases will most likely have a negative effect on the more open vegetation types such as grass and lichen communities. These more open communities may probably face greater difficulties in handling 'extreme' events such as drier periods. The more dense plant communities of dwarf shrubs and mosses, while experiencing changes in temperature and soil moisture through experimental manipulation, showed no detectable response during this study. This indicates that initial responses might be low. Vegetation development along the Antarctic Peninsula will depend on the gradual amelioration of local conditions through increasing mean temperatures, while changes in the frequency or magnitude of extreme climatic events and the dispersal barriers for vascular plants and cryptogams may prove to be a greater barrier for new species settlement.

## Methods

### Study sites

**Figure 13:**
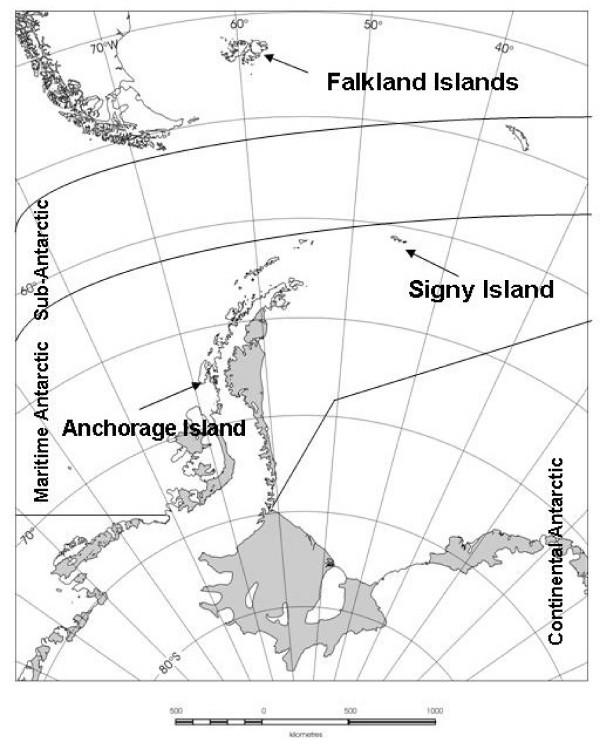
Map of the three field locations.

The study took place at the Falkland Islands (51°76'S 59°03'W), Signy Island (60°71'S 45°59'W) and Anchorage Island (67°61'S 68°22'W) (Fig. 13) over a period of 3 growing seasons between 2003 and 2006. Experimental manipulations were installed in coastal communities in two dominant vegetation types available at the three locations. We explicitly chose coastal communities, as those are the only extensive macroscopic vegetation types present in the Antarctic Peninsula region. As a result these were inevitably different at the Falklands and the two Maritime Antarctic locations. The sites selected included moss or lichen dominated communities on the Maritime Antarctic islands and grass or dwarf shrub communities on the Falkland Islands [2,45]. The communities on each island lie less than 100 m apart.

The Falkland Islands vegetation is dominated by grasses and dwarf shrubs, due to exposure to typically high winds and low precipitation [45]. The Falkland Islands do not experience prolonged periods with snow cover. The study site here was at Saladero Farm, south-west of Brenton Loch, near the settlement of Goose Green on East Falkland (51°7567'S 59°0298'W). The two communities selected for sampling and manipulation were dwarf shrub dominated vegetation (dominance of *Empetrum rubrum *Vahl ex Willd.) and a rocky, grass dominated, vegetation (co-dominance of *Festuca magellanica *Lam., *Poa annua *L. and *P. pratensis *L.). The two communities are on a small peninsula on the shore of Brenton Loch, 1–2 m above sea level. The dwarf shrub community grows on a 30–50 cm layer of peat. A very thin layer of soil on top of the rocky base layer, mainly sandstone, underlies the grass community.

Signy Island is a small (10 km^2^) island, within the northern Maritime Antarctic South Orkney Islands (60°71'S 45°59'W). The island has an ice cap giving rise to glaciers flowing towards the sea. During the summer months, December – February, up to c. 50% of the island's area becomes free of snow and ice, and has a well developed moss or lichen vegetation [46]. The study site on Signy Island was on the north facing 'back slope' area, near to the British Antarctic Survey (BAS) Signy Research Station. Where moss was present at this site, this community was dominated by *Polytrichum strictum *Brid. and *Chorisodontium aciphyllum *(Hook. f. & Wils.) Broth in Engl. The moss had a depth of approximately 20 cm underlain by a base layer of quartz-mica-schist [47]. The lichen community was present on a substratum of a similar rock type but did not develop complete vegetation cover while more weathering of the basal layer had occurred. It was dominated by *Usnea antarctica *Du Rietz.

Anchorage Island lies in Marguerite Bay (southern Maritime Antarctic) south of the BAS Rothera Research Station (67°61'S 68°22'W). The island is 2.5 km long and 500 m wide and is partly covered by semi-permanent snow and ice fields, although recently these have been decreasing rapidly in extent [48]. The island includes several rocky ridges and reaches a maximum height of 57 m asl. On the slopes of these ridges, there are patches of the moss *Sanionia uncinata *(Hedw.) Loeske and the grass *Deschampsia antarctica *Desv. However, the dominant vegetation consists of lichens, with *Usnea antarctica *being most prominent. The communities chosen for sampling and manipulation were dominated by *Sanionia uncinata *or by *Usnea antarctica*. The former consisted of patches (2–4 m^2^) of complete moss coverage located between rocks. A layer of dead moss of 0–10 cm underlies this vegetation. The lichen-dominated community consisted of bare rock and boulders with a partial coverage of *U. antarctica *and other lichen species.

### Experimental warming with Open Top Chambers

At each site, Open Top Chambers (OTCs) (Fig. 1) were placed on the soil surface to raise the air and soil temperature. The structure was based on the ITEX six sided model used extensively in Arctic climate manipulation studies [23,49,50]. OTCs were 0.5 m high and measured 1.8 m from opposite corners and 1.6 m from opposite sides at the top. For each community on Signy and Anchorage Islands, 6 plots of 2 × 4 m were chosen based on the visual similarity of vegetation. At the Falkland Islands, 9 plots were selected in the dwarf shrub community and, due to practical constraints, only 3 plots in the grass community. Each of these plots was divided into two sections, one in which the OTC was installed, and a neighbouring section acting as the control plot for that specific OTC, as in a split plot design. The placement of OTC and control plots was made randomly to avoid any possible consistent effects of OTCs on the neighbouring control plots by wind or snow. However, we were not able to measure wind speeds and snow accumulation during winter in and around the plots as the sites were not accessible.

### Environmental data recording

Environmental monitoring was undertaken at each of the three study locations, with sensors placed in three paired plots of each community. Air temperature at 5 cm, soil moisture (Water Content Reflectometer CS616, Campbell Scientific UK), air humidity (HMP45C Campbell Scientific UK) and photosynthetically active radiation (PAR) (SKP215 Campbell Scientific UK) were recorded every hour for the duration of the experiment. Due to the nature of the substratum, we were unable to place a Water Content Reflectometer to measure soil moisture in the grass community on the Falkland Islands. Therefore, this was determined gravimetrically, on one occasion during the summer of 2005 to indicate whether any differences existed between OTCs and control plots in this community. We did not measure this in the lichen community at Anchorage Island as the lichens were growing on rocks. A self-registering heated precipitation gauge (PLUVIO, OTT Hydrometrie) was installed at each location, to compare the yearly amount of precipitation with that of long-term means. At the three islands these are: 575, 400 and 500 mm y^-1 ^for the Falkland Islands, Signy and Anchorage Island respectively [51,52]. However, we did not obtain a reliable figure due to technical problems with maintaining sufficient battery power for the gauge on Signy and Anchorage Island.

### Vegetation recording

The abundance of higher plants and cryptogams was estimated using the point-intercept method [53]. We used a square frame of 30 × 30 cm with holes every 2.5 cm through which a pin (4 mm diameter) could be inserted vertically until it touched the ground. A "hit" was recorded when the pin touched a part of a plant, with a maximum of 10 hits per plant per pin. Moss and lichen species were recorded as present or absent for each point in the frame. Because more than one species could be hit by each pin, the sum of vegetation cover could exceed 100%. These measurements were made during three consecutive field seasons. The vegetation at the Falkland Islands field sites was identified following Moore [45], and that of Signy and Anchorage Islands using Ochyra [54] and Bednarek-Ochyra *et al. *[55]. Lichen identifications were confirmed by D. Øvstedal and R. Lewis Smith. During each season of the study, the Falkland Islands were visited during November, Signy Island during December, and Anchorage Island between late January and early February.

### Statistical analyses

During the measuring period from November 2003 to February 2006 there were occasional gaps in the data set obtained due to technical problems. Therefore we used environmental data collected between December 2004 and November 2005, as this provided a complete year-round dataset for all three sites. The patterns observed over the duration of the sampling were similar to the period used for analyses. The existence of differences in environmental data between communities was examined using a repeated-measures ANOVA between the six communities with treatment (OTC vs. control plots) within a plot as a within-subject factor. Analyses were completed using annual means and seasonal (summer; Dec-Feb, autumn; Mar-May, winter; Jun-Aug and spring; Sep-Nov) means. Log transformations were applied where appropriate to reduce the variance of the residuals. However, for the temperature data, this transformation was inappropriate, and the non-homogeneity of variances could not be resolved. Therefore, data obtained from the Falkland Islands communities was tested separately from that obtained from the four Maritime Antarctic communities. Post-hoc (Tukey HSD) tests were used to test for differences between communities. To identify any relationship between soil moisture and soil temperature a linear regression model was applied. Analyses were completed using the package Brodgar 2.5 and Statistica.

Diversity was estimated using Shannon's diversity index (H), calculated as:

H=−∑i=1Spiln⁡pi
 MathType@MTEF@5@5@+=feaagaart1ev2aaatCvAUfKttLearuWrP9MDH5MBPbIqV92AaeXatLxBI9gBaebbnrfifHhDYfgasaacPC6xNi=xI8qiVKYPFjYdHaVhbbf9v8qqaqFr0xc9vqFj0dXdbba91qpepeI8k8fiI+fsY=rqGqVepae9pg0db9vqaiVgFr0xfr=xfr=xc9adbaqaaeGacaGaaiaabeqaaeqabiWaaaGcbaGaemisaGKaeyypa0JaeyOeI0YaaabCaeaacqWGWbaCdaWgaaWcbaGaemyAaKgabeaakiGbcYgaSjabc6gaUjabdchaWnaaBaaaleaacqWGPbqAaeqaaaqaaiabdMgaPjabg2da9iabigdaXaqaaiabdofatbqdcqGHris5aaaa@3E94@

Where H = Shannon's diversity index, S = total number of species in the community (richness) and P = proportion of *S *made up of the *i*th species.

Evenness (E) was calculated by dividing the H by the natural logarithm of the number of species.

The diversity indices obtained across community types and treatments were also analysed using repeated measures ANOVA. For the analyses of diversity change after two years, the individual plot diversity index obtained in 2003/04 was subtracted from that obtained in 2005/06 to give a measure of the net change in diversity (if any). Total vegetation cover (calculated as percentage of point intercept hits with plants of the total number (121) of points in the quadrant) total number of hits with change was analysed in a similar way for the difference between 2003/04 and 2005/06. However, again there was non-homogeneity of variance in the data that could not be resolved by a mathematical transformation. Therefore, a one-way ANOVA between the control and OTC plots was employed to analyse the change in cover, separately for each community. The differences in total hits per plot obtained for each species between 2003/04 and 2005/06 were also analysed using a repeated measures ANOVA as described above.

## Authors' contributions

SB set up the study sites, carried out field sampling and performed statistical analyses. AH contributed to the setting up of the study sites, participated in its design and coordination and helped to draft the manuscript. PC and RA participated in the design and coordination and helped to draft the manuscript.
